# Mortality rates from asbestos-related diseases in Italy during the first year of the COVID-19 pandemic

**DOI:** 10.3389/fpubh.2023.1243261

**Published:** 2024-01-16

**Authors:** Lucia Fazzo, Enrico Grande, Amerigo Zona, Giada Minelli, Roberta Crialesi, Ivano Iavarone, Francesco Grippo

**Affiliations:** ^1^Department of Environment and Health, Istituto Superiore di Sanità, Rome, Italy; ^2^World Health Organization Collaborating Centre for Environmental Health in Contaminated Sites, Rome, Italy; ^3^Integrated System for Health, Social Assistance and Welfare, Italian National Institute of Statistics, Rome, Italy; ^4^Statistical Service, Istituto Superiore di Sanità, Rome, Italy

**Keywords:** COVID-19, asbestos, mesothelioma, asbestosis, mortality

## Abstract

**Background and aim:**

Patients with interstitial lung diseases, including asbestosis, showed high susceptibility to the SARS-CoV-2 virus and a high risk of severe COVID-19 symptoms. Italy, highly impacted by asbestos-related diseases, in 2020 was among the European countries with the highest number of COVID-19 cases. The mortality related to malignant mesotheliomas and asbestosis in 2020 and its relationship with COVID-19 in Italy are investigated.

**Methods:**

All death certificates involving malignant mesotheliomas or asbestosis in 2010–2020 and those involving COVID-19 in 2020 were retrieved from the National Registry of Causes of Death. Annual mortality rates and rate ratios (RRs) of 2020 and 2010–2014 compared to 2015–2019 were calculated. The association between malignant pleural mesothelioma (MPM) and asbestosis with COVID-19 in deceased adults ≥80 years old was evaluated through a logistic regression analysis (odds ratios: ORs), using MPM and asbestosis deaths COVID-19-free as the reference group. The hospitalization for asbestosis in 2010–2020, based on National Hospital Discharge Database, was analyzed.

**Results:**

In 2020, 746,343 people died; out of them, 1,348 involved MPM and 286 involved asbestosis. Compared to the period 2015–2019, the mortality involving the two diseases decreased in age groups below 80 years; meanwhile, an increasing trend was observed in subjects aged 80 years and older, with a relative mortality risks of 1.10 for MPM and 1.17 for asbestosis. In subjects aged ≥80 years, deaths with COVID-19 were less likely to have MPM in both genders (men: OR = 0.22; women: OR = 0.44), while no departure was observed for asbestosis. A decrease in hospitalization in 2020 with respect to those in 2010–2019 in all age groups, both considering asbestosis as the primary or secondary diagnosis, was observed.

**Conclusions:**

The increasing mortality involving asbestosis and, even if of slight entity, MPM, observed in people aged over 80 years during the 1^st^ year of the COVID-19 pandemic, aligned in part with the previous temporal trend, could be due to several factors. Although no positive association with COVID-19 mortality was observed, the decrease in hospitalizations for asbestosis among individuals aged over 80 years, coupled with the increase in deaths, highlights the importance of enhancing home-based assistance during the pandemic periods for vulnerable patients with asbestos-related conditions.

## Introduction

During the COVID-19 pandemic, it was observed that patients with comorbidities, including chronic lung diseases, tended to have more severe symptoms of COVID-19, with more complications.^1^

Among chronic pulmonary diseases, interstitial lung diseases (ILDs) have been particularly focused. These diseases comprise a broad and heterogeneous spectrum of pulmonary parenchymal disorders of known and unknown causes, for example, idiopathic pulmonary fibrosis (IPF) and acute interstitial pneumonia. ILDs can appear as a manifestation of an underlying systemic illness and can also result from occupational, environmental, or drug exposures (1). All ILDs, especially IPF, are characterized by acute exacerbations, associated with thoracic surgical procedures and viral infections (2), with a particularly high mortality rate (35%−70%).

A systematic review and quantitative meta-analysis evaluated the relationship between pre-existing ILDs and outcomes of COVID-19. The prevalence of ILDs in COVID-19 patients across the globe was estimated as 1.4%, which was significantly higher in non-surviving COVID-19 patients, and the mortality rate was twice as higher in patients with ILDs than those without ILDs, suggesting that ILDs are associated with poor outcomes of COVID-19 (3). Drake et al. investigated 161 international and multicentre subjects across Europe with a previous diagnosis of ILD and 322 subjects without the diagnosis, admitted to the hospital with COVID-19 in March-April 2020: mortality in patients with ILD was significantly higher than mortality in those without ILD. ILD was associated with a 60% increase in the risk of death (2). ILDs were reported as a significant risk factor for COVID-19 in adults with COVID-19 admitted in 26 Turkish centers (4) and in the population cohort studies in England (5) and Korea (6).

Asbestosis is a specific diffuse ILD due to high occupational exposure to asbestos fibers. Pulmonary function anomalies can include gas exchange abnormalities, restrictive patterns, and obstructive features due to small airway disease (7).

Italy was one of the main producers of asbestos among the European countries, up to the 1992 ban, and it still remains among the countries most impacted by asbestos-related diseases (ARDs), such as asbestosis and malignant mesotheliomas (MM) (8).

MM is among the diseases considered by the Thoracic Centres International Coronavirus Disease 2019 Collaboration Registry (TERAVOLT) (9–13), reporting a high COVID-19 mortality rate in patients with thoracic malignancies (11). Lung cancer may not appear as an extremely high risk factor for susceptibility to COVID-19, at least not in the same way as cardiovascular diseases, chronic obstructive pulmonary diseases, and diabetes; however, patients with lung cancer, when affected, have an increased risk of experiencing a more severe illness (11). These data confirm the first observation that, based on a sample of 102 patients, the COVID-19 course is more severe in patients with lung cancer (13). MM was among the first four comorbidities significantly associated with an increase in COVID-19 incidence, shown by a geographical clustering analysis carried out in US counties. The authors suggest that protecting subjects from diabetes, tuberculosis, mesothelioma, cardiomyopathy, and myocarditis can help reduce COVID-19 mortality (14).

Italy was the first European country to face the SARS-CoV-2 epidemic. The World Health Organization (WHO) indicated that Italy was one of the European countries with the highest number of newly reported deaths associated with COVID-19 (15), and it estimated that Italy experienced an exceeding mortality of 100,431 deaths in 2020 (16).

Grande et al. reported that, in Italy, from March to April 2020, all-cause mortality rates increased by 39% in men and 31% in women with respect to the same months in 2015–2019. COVID-19 was the leading cause of death primarily among men and the second among women; in addition, remarkable increases in mortality rates were reported for influenza and pneumonia, followed by diabetes, hypertensive diseases, dementia, and Alzheimer's disease; the mortality rates for neoplasms showed a slight decrease (17). In 2021, a study on the pathological patterns of the individuals deceased from COVID-19 in Italy, based on the National Survey of COVID-19 positive individuals, reported neoplasms, hypertensive heart diseases, and diabetes as the most frequently mentioned comorbidities during the two considered periods of the COVID-19 pandemic (February–April and May–September 2020) (18). During the first wave (March–May 2020), the geographical spread of the pandemic was heterogeneous. In Southern Regions and the islands, the infection's spread was limited; in Central Regions, it was, on average, higher than in the South, while the spread of the virus was high in Northern Regions (19). Subsequently, in the summer of 2020, the spread of the virus was very limited; however, by the end of September, an increasing number of outbreaks affected the entire country, marking the beginning of the second wave. The first wave mainly affected Northern Italy, whereas the second wave involved the country from North to South (20).

The present study aims to analyse the mortality rates in the Italian general population from MM (especially pleural) and asbestosis, during the 1^st^ year of the COVID-19 pandemic, and the possible association of these causes with COVID-19 mortality.

## Materials and methods

### Study design

The present study is a cross-sectional study of deaths related to asbestosis and MM. This study also explores the association of these diseases with COVID-19.

### Settings

Mortality data are derived from the National Register of Causes of Death (RCoD) managed by the Italian National Institute of Statistics to which all death certificates issued must be referred by law. At the beginning of the present investigation, the most recent available data refer to 2020. The hospitalization data are derived from the National Hospital Discharge Database (NHDB) of the Ministry of Health, which archives regional data from any Italian public and private hospital after an urgent or planned (diagnostic or interventional) admission.

### Participants

The study analyzed all Italian deaths involving asbestosis, MM, and COVID-19 recorded from 2010 to 2020 as well as all Italian hospitalizations related to asbestosis, during the same period.

### Outcomes

The age-standardized mortality rates (SRs) by disease and odds ratios (ORs) for having vs. not having COVID-19 among MPM or asbestosis deaths over 80 years old in 2020 were calculated. The hospitalization SR for asbestosis was calculated in the 2010–2020 period.

### Data sources

The medical certificate of the causes of death includes two parts: in part 1, the certifying physician reports the chain of diseases or events leading to death; and in part 2, he/she reports other relevant conditions contributing to death but not directly responsible for it. The RCoD data include the underlying cause of death (i.e., the disease or injury which initiated the train of events directly leading to death), and all diseases, conditions, or events listed in the death certificate (multiple causes of death), classified according to the International Classification of Diseases 10th revision, ICD-10.

The following ICD-10 codes were considered: J61, pneumoconiosis due to asbestos and other mineral fibers and C45.0–C45.9 for MM, with the code C45.0 specific for malignant pleural mesothelioma (MPM). Deaths due to MM (at any site), MPM, or asbestosis were identified considering the underlying cause of death. Deaths involving the two diseases were defined as deaths mentioning them anywhere on the death certificate, i.e., among the multiple-cause codes (thus not considering the underlying cause code only). Deaths involving COVID-19 were identified by selecting codes U07.1-U07.2 among multiple-cause codes. For the regression analysis, deaths with the ICD-10 code J84.9 anywhere in the certificate were excluded, considering that some COVID-19 cases were certified as “interstitial pneumonia.”

In NHDB, for each patient, demographic data (e.g., gender, date of birth, place of residence), as well as the primary diagnosis and up to five secondary discharge diagnoses, are recorded; diagnoses are codified according to the International Classification of Diseases, Ninth Revision, Clinical Modification (ICD-9-CM). The first admission with the presence of asbestosis (ICD-9-CM: 501) was considered for all hospital discharge diagnoses during the study period (2010–2020).

### Analyses

Directly age-standardized mortality rates for the overall population resident in Italy were calculated for deaths due to MM, MPM, or asbestosis (underlying cause) and for deaths involving these diseases (multiple-causes), using 5-year age-specific mortality rates with the oldest age group being 95 years and older and the European population as the standard.^2^

The rates, using the mid-year resident population as denominators, were calculated for each year from 2010 to 2020 and for the period of 2015–2019 for the overall population and for separate age-groups (<50 years, 50–64 years, 65–79 years, ≥80 years). Mortality rate ratios (RRs) of 2020 compared to 2015–2019 were calculated, with a 90% confidence interval (CI) (21). In addition, RRs of 2010–2014 vs. 2015–2019 were also calculated to estimate the previous temporal trend.

Considering that approximately 80% of all MM are located in pleura and the similarities with COVID-19 manifestations, analyses focused on MPM deaths.

To investigate the association between COVID-19 and MPM or asbestosis, a sex-stratified logistic regression model, adjusting for 5-year age groups, was applied to the mortality rate of 2020 for the age group of ≥80 years. ORs between deaths involving MPM or asbestosis with the mention of COVID-19 and those without COVID-19 were calculated, considering the latter group as reference. We mainly focused on multiple causes of deaths since they allow us to investigate the burden of mortality involving the diseases under the study independently from the rules of underlying cause selection and limit possible underestimation. Specifically, in 2020, some causes could have been underreported in COVID-19 deaths. In addition, the multiple causes data allow us to study the association between comorbidities in the death event.

Similar to mortality analyses, annual SRs of hospitalization in the 2010–2020 period were calculated, considering all diagnoses (both primary and secondary) in the hospital discharges. RRs of 2020 and SRs of 2010–2014, with respect to the annual mean of 2015–2019, were calculated by sex and age group to investigate the temporal trend.

## Results

In Italy, 746,324 people died in 2020; out of them, 74 subjects deceased from asbestosis, 1,552 subjects deceased from MM (including 1,249 subjects deceased from MPM), and 78,673 subjects deceased from COIVD-19. Considering the multiple causes of death, 286 subjects deceased with asbestosis and 1,695 subjects with MM (including 1,348 subjects deceased with MPM) (Table 1). The deaths for all causes and those due to COVID-19 in 2020 and the percentage of variation of all-cause mortality compared to the previous periods are shown in Supplementary Table S1. The differences in deaths due to or involving mesothelioma or asbestosis, which is particularly remarkable for asbestosis (Table 1) because of the low lethality of the disease, are in agreement with the literature (8).

**Table 1:** Deaths underlying and involving all malignant mesothelioma (MM), malignant pleural mesothelioma (MPM), and asbestosis, by age class and sex.

	**Due to (underlying cause)**		**Involving (multiple causes)**	
	**Males**	**Females**	**Males**	**Females**
**MM**				
0–49	7	3	7	3
50–64	102	58	110	61
65–79	590	176	642	188
≥80	420	196	470	214
Total	1,119	433	1,229	466
**MPM**				
0–49	5	3	5	3
50–64	76	38	82	40
65–79	482	140	516	150
≥80	350	155	385	167
Total	913	336	988	360
**Asbestosis**				
0–49	1	0	1	0
50–64	0	0	5	1
65–79	18	0	90	5
≥80	54	1	173	11
Total	73	1	269	17

Italy, 2020.

Supplementary Table S2 shows the number of deaths due to and involving MM, MPM, and asbestosis, by sex, age group, and year, for the period of 2010–2020.

The numbers of deaths involving MPM and asbestosis and age-standardized rates (per 100,000 inhabitants) in the 2010–2020 period by year and sex are reported in Table 2. In 2014 and 2015, both diseases showed a peak of SR, and a slight decrease in MPM was observed in 2019 compared to 2018. In 2020, the number of deaths involving asbestosis increased by 13% compared to the 5-year previous period (mean average of 2015–2019); meanwhile, the mortality involving MPM showed a slight decrease (1,348 MPM deaths, a combination of men and women, in 2020 vs. 1,373 mean average in 2015–2019) (Table 2).

**Table 2:** Mortality involving malignant pleural mesothelioma (MPM) and asbestosis, by sex and year.

**Year**	**MPM**					**Asbestosis**				
	**Deaths**		**SR**			**Deaths**		**SR**		
	**M**	**F**	**M**	**F**	**Total**	**M**	**F**	**M**	**F**	**Total**
2010	839	345	3.08	0.96	1.88	192	10	0.76	0.02	0.31
2011	825	341	3.00	0.93	1.83	198	14	0.77	0.03	0.32
2012	923	357	3.33	0.97	1.98	219	21	0.84	0.05	0.35
2013	970	332	3.44	0.87	1.98	225	20	0.83	0.05	0.36
2014	1,059	371	3.64	0.96	2.12	239	19	0.86	0.04	0.37
2015	1,011	390	3.42	1.01	2.05	250	15	0.89	0.03	0.37
2016	1,024	358	3.41	0.90	1.99	232	29	0.81	0.06	0.36
2017	1,017	326	3.32	0.80	1.90	229	16	0.78	0.03	0.32
2018	1,016	404	3.25	0.98	1.97	228	18	0.76	0.04	0.32
2019	939	377	2.97	0.89	1.79	234	17	0.76	0.03	0.32
2020	988	360	3.10	0.85	1.83	269	17	0.86	0.04	0.36

Deaths and standardized rate (per 100,000 inhabitants). Italy, 2010–2020. M, males; F, females; SR, standardized rates.

**Figure 1:**
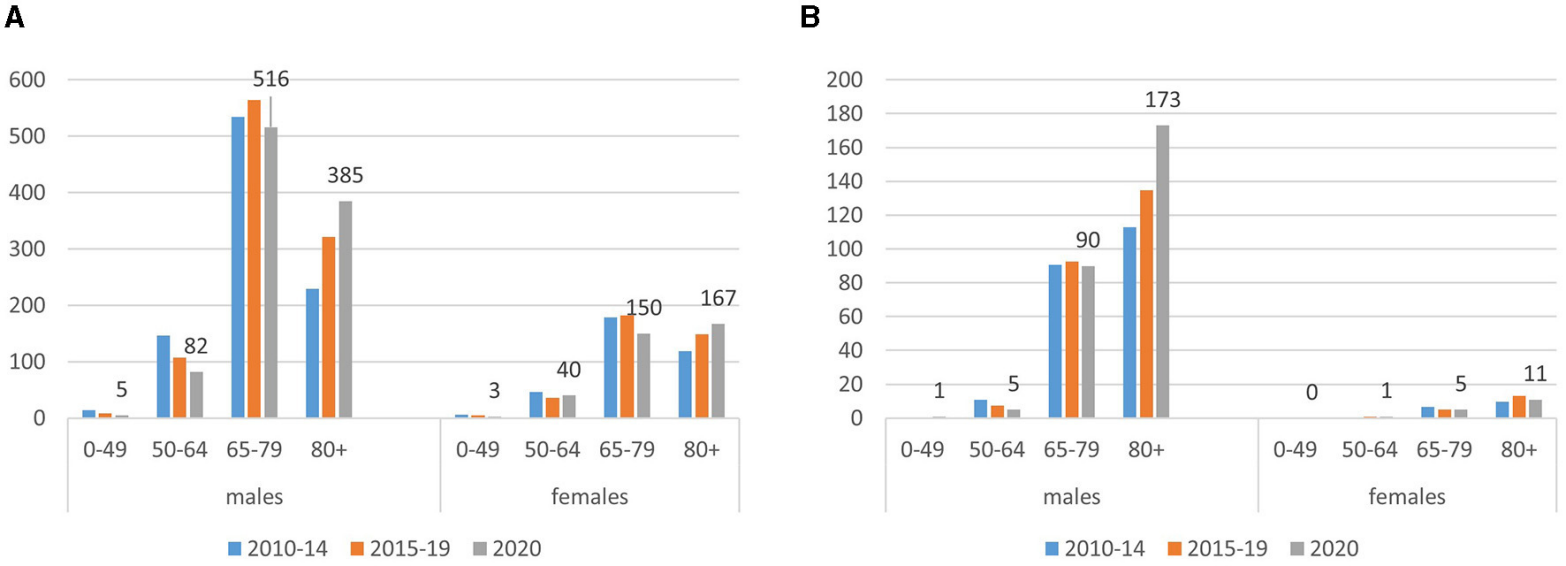
Number of deaths involving malignant pleural mesothelioma [MPM: **(A)**] and asbestosis **(B)**, by sex and age group. Italy, 2020. Deaths and the average annual number of deaths observed in 2010–2014 and 2015–2019.

Figure 1 shows the number of subjects deceased with MPM and asbestosis in 2020 compared to the annual average of the previous periods (2010–2014 and 2015–2019) by sex and age group. The great majority of deaths with both MPM and asbestosis occurred in the male population and in the age group of 65–79 years and over 80 years, respectively. Women younger than 50 years deceased with asbestosis were not observed. In 2020, an increase in the number of deaths involving MPM and asbestosis in men over 80 years, particularly, was observed.

The SRs of mortality for MPM and asbestosis, considered as involving and the underlying causes of death, by age group and sex along rate ratios (RRs) in 2020 and 2010–2014, compared to 2015–2019, are reported in Table 3 and Supplementary Table S3, respectively.

**Table 3:** Mortality involving malignant pleural mesothelioma (MPM) and asbestosis.

**Age group**	**Deaths**			**Standardized rate**			**Rate ratio (90% CI)**		
	**M**	**F**	**Total**	**M**	**F**	**Total**	**M**	**F**	**Total**
**MPM**									
**2010–2014**									
0–49	68	28	96	0.07	0.03	0.05	1.51 (1.1–2.07)	1.29 (0.81–2.06)	1.43 (1.1–1.87)
50–64	732	230	962	2.56	0.76	1.63	1.44 (1.32–1.59)	1.38 (1.17–1.62)	1.43 (1.32–1.55)
65–79	2,672	892	3,564	13.21	3.63	7.98	1.02 (0.97–1.06)	1.03 (0.95–1.11)	1.01 (0.98–1.05)
≥80	1,144	596	1,740	17.13	4.91	9.19	0.85 (0.79–0.9)	0.87 (0.79–0.95)	0.83 (0.79–0.87)
Total	4,616	1,746	6,362	3.31	0.94	1.96	1.01 (0.98–1.05)	1.02 (1.01–1.08)	1.01 (0.98–1.04)
**2015–2019**									
0–49	44	22	66	0.04	0.02	0.03	1	1	1
50–64	536	179	715	1.77	0.55	1.14	1	1	1
65–79	2,822	909	3,731	13.00	3.54	7.87	1	1	1
≥80	1,605	745	2,350	20.24	5.65	11.11	1	1	1
Total	5,007	1,855	6,862	3.27	0.92	1.94	1	1	1
**2020**									
0–49	5	3	8	0.03	0.02	0.02	0.59 (0.27–1.28)	0.73 (0.26–2.01)	0.64 (0.34–1.18)
50–64	82	40	122	1.29	0.59	0.93	0.73 (0.6–0.89)	1.07 (0.8–1.43)	0.81 (0.69–0.96)
65–79	516	150	666	11.84	2.94	7.02	0.91 (0.84–0.98)	0.83 (0.72–0.96)	0.89 (0.83–0.96)
≥80	385	167	552	22.25	6.03	12.27	1.10 (1.00–1.21)	1.07 (0.93–1.23)	1.10 (1.02–1.19)
Total	988	360	1,348	3.10	0.85	1.83	0.95 (0.89–1)	0.93 (0.91–1.03)	0.94 (0.9–0.99)
**Asbestosis**									
**2010–2014**									
0–49	0	0	0	0	0	0			
50–64	54	2	56	0.19	0.01	0.09	1.56 (1.1–2.22)	0.54 (0.13–2.24)	1.46 (1.04–2.05)
65–79	454	34	488	2.25	0.14	1.09	1.05 (0.94–1.17)	1.38 (0.9–2.13)	1.05 (0.95–1.17)
≥80	565	48	613	9.03	0.39	3.29	0.97 (0.88–1.07)	0.82 (0.6–1.12)	0.93 (0.85–1.02)
Total	1,073	84	1,157	0.81	0.04	0.34	1.02 (0.95–1.09)	1 (0.99–1.29)	1.01 (0.94–1.08)
**2015–2019**									
0–49	0	0	0	0	0	0	1	1	1
50–64	36	4	40	0.12	0.01	0.06	1	1	1
65–79	463	26	489	2.14	0.10	1.03	1	1	1
≥80	674	65	739	9.30	0.48	3.53	1	1	1
Total	1,173	95	1,268	0.80	0.04	0.34	1	1	1
**2020**									
0–49	1	0	1	0.01	0	0			
50–64	5	1	6	0.08	0.02	0.05	0.64 (0.29–1.4)	1.2 (0.19–7.53)	0.7 (0.34–1.44)
65–79	90	5	95	2.07	0.10	1.00	0.97 (0.8–1.17)	0.97 (0.44–2.18)	0.97 (0.81–1.17)
≥80	173	11	184	10.74	0.39	4.13	1.16 (1–1.33)	0.81 (0.47–1.39)	1.17 (1.02–1.34)
Total	269	17	286	0.86	0.04	0.36	1.07 (0.96–1.2)	0.89 (0.87–1.39)	1.07 (0.96–1.19)

Standardized rates (per 100,000 inhabitants) and rate ratios of 2020 and 2010–2014 compared to 2015–2019. M, males; F, females.

A slightly increasing temporal trend over the entire study period was observed in the population over 80 years. In comparison with the 2015–2019 period, while the 2020 mortality in the overall population is similar for both diseases, it increased by 10% for MPM (RR = 1.10, 90% CI: 1.02–1.19) and by 17% for asbestosis (RR = 1.17, 90%CI: 1.02–1.34), in the age group over 80 years (Table 3).

Table 4 shows the results of the logistic model. Compared to deaths without any mention of COVID-19 (reference), deaths involving COVID-19 have a significantly lower probability of presenting MPM among causes of death in both men and women (men: OR = 0.22; women: OR = 0.44), while no departure was observed for asbestosis.

**Table 4:** Odds ratios of malignant pleural mesothelioma (MPM) and asbestosis mentioned in death certificates, according to the presence of COVID-19.

	**All deaths**		**MPM**				**Asbestosis**			
	**Males**	**Females**	**Males**		**Females**		**Males**		**Females**	
	***n***	***n***	***n***	**OR (90% CI)**	***n***	**OR (90% CI)**	***n***	**OR (90% CI)**	***n***	**OR (90% CI)**
Deaths without COVID-19	173,124	252,352	371	1	158	1	150	1	10	1
Deaths with COVID-19	26,378	30,687	14	0.22 (0.14–0.35)	9	0.44 (0.25–0.77)	23	0.96 (0.66–1.39)	1	0.79 (0.14–4.45)

Age of ≥ 80 years. Italy, 2020. n, number of deaths; OR, odds ratio; CI, confidence interval.

Hospitalization from asbestosis showed an overall decreasing temporal trend in 2020 with respect to the annual mean of 2015–2019 and 2010–2014, in all age groups, both considering asbestosis as primary or secondary diagnoses (Table 5).

**Table 5:** Hospitalizations with asbestosis in primary diagnosis only and in all diagnoses.

**Age group**	**Hospitalized**			**Standardized rate**			**Rate ratio (90% CI)**		
	**M**	**F**	**Total**	**M**	**F**	**Total**	**M**	**F**	**Total**
**Primary diagnosis only**									
**2010–2014**									
0–49	18	1	19	0.02	0.00	0.01	18.21 (2.43–136.43)	0.26 (0.03–2.36)	3.39 (1.25–9.15)
50–64	171	7	178	0.59	0.02	0.30	3.75 (2.72–5.16)	1.86 (0.54–6.34)	3.60 (2.64–4.91)
65–79	373	21	394	1.82	0.08	0.88	1.90 (1.60–2.25)	3.69 (1.49–9.17)	1.93 (1.64–2.28)
≥80	120	8	128	1.71	0.07	0.66	1.57 (1.18–2.07)	0.74 (0.30–1.82)	1.40 (1.08–1.82)
Total	682	37	719	0.48	0.02	0.22	2.11 (1.86–2.41)	1.61 (0.96–2.69)	2.09 (1.84–2.37)
**2015–2019**									
0–49	1	4	5	0.00	0.00	0.00	1	1	1
50–64	48	4	52	0.16	0.01	0.08	1	1	1
65–79	208	6	214	0.96	0.02	0.46	1	1	1
≥80	88	12	100	1.09	0.09	0.47	1	1	1
Total	345	26	371	0.23	0.01	0.11	1	1	1
**2020**									
0–49	1	0	1	0.00	0	0.00	5.11 (0.31–81.68)	0	0.89 (0.1–7.65)
50–64	1	0	1	0.02	0	0.01	0.10 (0.01–0.74)	0	0.09 (0.01–0.68)
65–79	22	3	25	0.50	0.06	0.26	0.52 (0.33–0.80)	2.51 (0.63–10.06)	0.57 (0.38–0.87)
≥80	10	0	10	0.55	0	0.22	0.51 (0.26–0.97)	0	0.46 (0.24–0.87)
Total	34	3	37	0.11	0.01	0.05	0.47 (0.32–0.67)	0.64 (0.19–2.14)	0.48 (0.34–0.68)
**All diagnoses**									
**2010–2014**									
0–49	33	4	37	0.03	0.00	0.02	2.31 (1.19–4.51)	0.60 (0.17–2.14)	1.73 (0.98–3.06)
50–64	416	25	441	1.45	0.08	0.74	2.18 (1.84–2.58)	1.24 (0.69–2.21)	2.09 (1.78–2.46)
65–79	1,679	113	1,792	8.22	0.46	3.97	1.34 (1.24–1.43)	1.72 (1.27–2.33)	1.34 (1.25–1.44)
≥80	897	75	972	13.39	0.62	5.11	1.01 (0.82–0.97)	0.82 (0.61–1.11)	0.97 (0.89–1.05)
Total	3,025	217	3,242	2.16	0.12	0.99	1.28 (1.21–1.35)	1.24 (1.02–1.51)	1.28 (1.22–1.35)
**2015–2019**									
0–49	12	6	18	0.01	0.01	0.01	1	1	1
50–64	199	21	220	0.66	0.07	0.36	1	1	1
65–79	1,332	68	1,400	6.16	0.26	2.97	1	1	1
≥80	1,010	99	1,109	13.22	0.75	5.29	1	1	1
Total	2,553	194	2,747	1.69	0.09	0.77	1	1	1
**2020**									
0–49	2	0	2	0.01	0.00	0.01	0.76 (0.17–3.42)	0	0.51 (0.12–2.20)
50–64	20	0	20	0.32	0.00	0.15	0.48 (0.30–0.75)	0	0.43 (0.27–0.68)
65–79	213	9	222	4.87	0.17	2.33	0.79 (0.68–0.91)	0.66 (0.33–1.32)	0.79 (0.68–0.90)
≥80	200	8	208	11.61	0.29	4.62	0.88 (0.75–1.02)	0.38 (0.19–0.79)	0.87 (0.75–1.01)
Total	435	17	452	1.36	0.04	0.60	0.80 (0.72–0.89)	0.43 (0.26–0.71)	0.78 (0.71–0.86)

Standardized rates (per 100,000 inhabitants) and rate ratios of 2020 and 2010–2014 compared to 2015–2019. M, males; F, females; CI, confidence interval.

## Discussion

Italy was the first European country that confronted the COVID-19 pandemic, with an uneven trend, particularly in 2020 (19). The differences across geographical areas may due to a number of factors, i.e., the high concentration of economic and commercial activities in Northern Italy and the different levels of air pollution that may have made the northern population more susceptible to respiratory infections (19, 22).

ILDs are considered among the comorbidities that could determine a higher risk of severe COVID-19, causing more severe symptoms and complications.

Some ARDs, such as MPM and asbestosis, affect the lungs, and asbestosis is included among the ILDs.

In Italy, approximately 4,400 deaths by year due to ARDs, including 1,515 deaths due to MM and 58 deaths due to asbestosis, have been estimated in the 2010–2016 period (8). The vulnerability to COVID-19 of the subjects affected by ARDs was of particular concern among subjects formerly exposed to asbestos and their relatives.

The present study investigated the MPM and asbestosis mortality, during the 1^st^ year of the COVID-19 pandemic, in Italy, using both underlying and multiple causes based on nationwide data. To the best of our knowledge, there are no specific studies on asbestosis mortality in relation to the COVID-19 pandemic.

The study is based on the use of nationwide data, which include all deaths that occurred in Italy. The causes of deaths are stated by physicians, and the coding procedures are highly standardized and internationally comparable, even if an underreporting or lack of specificity of the cause of death cannot be completely excluded. In particular, during the pandemic period, some causes could have been underreported in favor of COVID-19; meanwhile, in the first months of the pandemic, an underreporting of COVID-19 could have occurred (23, 24); and this could represent a limitation of the present investigation. However, a value of the study is the use of multiple causes of death data. Such data allow for a more comprehensive evaluation of mortality related to MPM and asbestosis in comparison with the use of death underlying cause data only. The replicability of the analyses presented in this study depends on the availability of routinely collected multiple causes-of-death data, which is globally increasing.

Considering multiple-cause data, we found that, in 2020, the mortality exceeded the 2015–2019 rate in both men and women over 80 years for MPM and among men for asbestosis. Similar trends were observed since 2010. These increases are slightly higher than the increase in mortality from all causes other than COVID-19 (+19% vs. +9%) (Supplementary Tables S1, S2).

The logistic regression analysis, performed on the age group of ≥80 years, highlights a significant negative association between COVID-19 and MPM or asbestosis in 2020 death certificates. These results suggest that the increase in mortality from such conditions might be only indirectly related to COVID-19, even if a possible underestimation of the risk could not be excluded, considering the possible under-diagnosis of COVID-19, particularly in the first wave of the pandemic (23, 24).

The results regarding mesothelioma in the overall population comply with previous studies, highlighting no increases in mortality from malignant tumors during the COVID-19 pandemic in Italy (17, 25). One of the reasons hypothesized was that patients with cancer were not at a higher risk of infection due to the protective effect of social distancing measures (26). Italy has a surveillance program for malignant mesothelioma cases, performed by the aforementioned ReNaM, a network with regional organizations. An Operational Center is established at each region that identifies all mesothelioma cases in its territory, analyzing the occupational, residential, and environmental history of sick individuals to identify asbestos exposure contexts. Our results are in agreement with the recent study based on ReNaM data, reporting that, in Italy, the restriction put in place to control the COVID-19 pandemic had little or no effect on the new MM diagnoses (27). In addition, the temporal trend of MPM mortality observed in 2010–2019 is in agreement with the previous models (28) and the more recent national MM mortality surveillance, reporting a slight decrease in 2019 (29).

The observed decrease in the hospitalizations for asbestosis reflects the reduction in all admissions observed in 2020. In Italy, there were 6.5 million hospitalizations in 2020, 22% lower than the average of the previous 3 years. This reduction was more pronounced during the first pandemic wave, where admission rates were 45% lower in April and 39% lower in May, compared to the average for the same months in 2017–2019. During the second wave, the effect on the hospital system was smaller.^3^

The reduction during the pandemic of healthcare service, including the health surveillance plans addressed to former asbestos exposure provided for by law,^4^ could have contributed to observed mortality increases. On the other hand, the increasing mortality involving asbestosis and malignant pleural mesothelioma observed particularly in people over 80 year of age could, almost in part, reflect the temporal trend that occurred since 2010 in the country and also due to the long latency period since the first exposure to asbestos of the two diseases.

## Conclusion

An increase in mortality was observed for asbestosis and, even if of slight entity, for MPM, in people aged over 80 years during the 1^st^ year of the COVID-19 pandemic. No direct association between these ARDs and COVID-19 mortality was observed; therefore, these diseases seem not to have been direct risk factors for COVID-19 mortality. The increase in 2020 of mortality rates for both diseases in ≥80-year old people could be, in part, explained by the increasing trend observed since 2010. The increase in mortality from asbestosis requires specific concern, considering that asbestosis is a non-lethal disease and that, in the same period, a huge decrease in the hospitalizations for this disease was observed in both genders. The reduction during the pandemic period of healthcare and hospitalization could be an indirect cause of the exceeding mortality. Further investigations, extending the analyses to the years after 2020, will provide a better understanding of what other factors may have determined the observed mortality trend and whether this has remained constant over time. Despite the limitations of the investigation, the results show that an increase in mortality is accompanied by a decrease in hospitalization, highlighting the importance of enhancing home-based assistance during the pandemic periods for vulnerable patients with asbestos-related morbidities.

## Data availability statement

The datasets presented in this article are not readily available because the analysis of the data used in this study complies with the European General Data Protection Regulation (EU GDPR 2016/679). The Italian Data Protection Authority authorized the processing of personal data relating to causes of death by Italian Institute of Statistics and to hospital discharge forms by Italian Institute for Health and other public institutions for reasons of public interest in public health. The analyses presented in the paper are based on aggregated data. Written consent for participation was not required for this study, in accordance with national legislation and institutional requirements. Requests to access the dataset for causes of death should be directed to Istat contact center (https://contact.istat.it/s/?language = it). Requests for access to the hospital discharge dataset should be directed at: GM, giada.minelli@iss.it.

## Author contributions

Conceptualization: LF. Methodology: LF, GM, II, FG, RC, and EG. Formal analysis: FG, EG, and GM. Writing—original draft preparation: LF, AZ, FG, and EG. Writing—review and editing: LF, FG, AZ, EG, RC, II, and GM. All authors have read and agreed to the published version of the manuscript.

## Footnotes

^1^ https://www.cdc.gov/coronavirus/2019-ncov/need-extra-precautions/people-with-medical-conditions.html

^2^ https://data.europa.eu/doi/10.2785/11470

^3^ https://www.agenas.gov.it/comunicazione/primo-piano/2112-agenas-e-istat-impatto-dell%E2%80%99epidemia-covid-19-sul-sistema-ospedaliero-italiano-anno-2020

^4^D.Lgs 81/80, https://www.gazzattaufficiale.it/eli/id/2008/04/30/008G0104/sg

## Supplementary material

Supplementary Table S1 Deaths from COVID-19 and other causes, Italy, 2010–2020.

Supplementary Table S2 Number of deaths due to and involving malignant mesothelioma and asbestosis, by age group, sex, and year. Italy, 2010–2020.

Supplementary Table S3 Mortality due to malignant pleural mesothelioma and asbestosis (underlying cause): standardized rates (per 100,000 inhabitants) and rate ratios of 2020 and 2010–2014 compared to 2015–2019.

## References

[1] Azadeh N Limper AH Carmona EM Ryu JH. The role of infection in interstitial lung diseases: a review. Chest. (2017) 152:842–52. 10.1016/j.chest.2017.03.033 28400116 PMC7094545

[2] Drake TM Docherty AB Harrison EM Quint JK Adamali H Agnew S. Outcome of hospitalization for COVID-19 in patients with interstitial lung disease. An international multicenter study. Am J Respir Crit Care Med. (2020) 202:1656–65. 10.1164/rccm.202007-2794OC 33007173 PMC7737581

[3] Ouyang L Gong J Yu M. Pre-existing interstitial lung disease in patients with coronavirus disease 2019: a meta-analysis. Int Immunopharmacol. (2021) 100:108145. 10.1016/j.intimp.2021.108145 34547678 PMC8450148

[4] Kokturk N Babayigit C Kul S Duru Cetinkaya P Atis Nayci S Argun Baris S. The predictors of COVID-19 mortality in a nationwide cohort of Turkish patients. Respir Med. (2021) 183:106433. 10.1016/j.rmed.2021.106433 33957434 PMC8079263

[5] Aveyard P Gao M Lindson N Hartmann-Boyce J Watkinson P Young D. Association between pre-existing respiratory disease and its treatment, and severe COVID-19: a population cohort study. Lancet Respir Med. (2021) 9:909–23. 10.1016/S2213-2600(21)00095-3 33812494 PMC8016404

[6] Lee H Choi H Yang B Lee SK Park TS Park DW. Interstitial lung disease increases susceptibility to and severity of COVID-19. Eur Respir J. (2021) 58:2004125. 10.1183/13993003.04125-2020 33888524 PMC8061231

[7] Wolff H Vehmas T Oksa P Rantanen J Vainio H. Asbestos, asbestosis, and cancer, the Helsinki criteria for diagnosis and attribution 2014: recommendations. Scand J Work Environ Health. (2015) 41:5–15. 10.5271/sjweh.3462 25299403

[8] Fazzo L Binazzi A Ferrante D Minelli G Consonni D Bauleo L. Burden of mortality from asbestos-related diseases in Italy. Int J Environ Res Public Health. (2021) 18:10012. 10.3390/ijerph181910012 34639316 PMC8508095

[9] Bestvina CM Whisenant JG Torri V Cortellini A Wakelee H Peters S. Coronavirus disease 2019 outcomes, patient vaccination status, and cancer-related delays during the omicron wave: a brief report from the TERAVOLT analysis. JTO Clin Res Rep. (2022) 3:100335. 10.1016/j.jtocrr.2022.100335 35619644 PMC9119707

[10] Whisenant JG Baena J Cortellini A Huang LC Lo Russo G Porcu L. A definitive prognostication system for patients with thoracic malignancies diagnosed with coronavirus disease 2019: an update from the TERAVOLT registry. J Thorac Oncol. (2022) 17:661–74. 10.1016/j.jtho.2021.12.015 35121086 PMC8804493

[11] Haineala B Zgura A Badiu DC Iliescu L Anghel RM Bacinschi XE. Lung cancer, COVID-19 infections and chemotherapy. In Vivo. (2021) 35:1877–80. 10.21873/invivo.12450 33910875 PMC8193347

[12] Garassino MC Whisenant JG Huang LC Trama A Torri V Agustoni F. COVID-19 in patients with thoracic malignancies (TERAVOLT): first results of an international, registry-based, cohort study. Lancet Oncol. (2020) 21:914–22. 10.1016/S1470-2045(20)30314-4 32539942 PMC7292610

[13] Luo J Rizvi H Preeshagul IR Egger JV Hoyos D Bandlamudi C. COVID-19 in patients with lung cancer. Ann Oncol. (2020) 31:1386–96. 10.1016/j.annonc.2020.06.007 32561401 PMC7297689

[14] Vahabi N Salehi M Duarte JD Mollalo A Michailidis G. County-level longitudinal clustering of COVID-19 mortality to incidence ratio in the United States. Sci Rep. (2021) 11:3088. 10.1038/s41598-021-82384-0 33542313 PMC7862666

[15] World Health Organization. Weekly epidemiological update on COVID-19. (2022). Available online at: https://www.who.int/publications/m/item/weekly-epidemiological-update-on-covid-19-−26-october-2022 (accessed March 13, 2023).

[16] World Health Organization. Weekly epidemiological update on COVID-19. (2022). Available online at: https://www.who.int/emergencies/diseases/novel-coronavirus-2019/situation-reports (accessed March 13, 2023).

[17] Grande E Fedeli U Pappagallo M Crialesi R Marchetti S Minelli G. Variation in cause-specific mortality rates in Italy during the first wave of the COVID-19 pandemic: a study based on nationwide data. Int J Environ Public Health. (2022) 19:805. 10.3390/ijerph19020805 35055627 PMC8776013

[18] Grippo F Grande E Maraschini A Navarra S Pappagallo M Marchetti S. Evolution of pathology patterns in persons who died from COVID-19 in Italy: a national study based on death certificates. Front Med. (2021) 8:645543. 10.3389/fmed.2021.645543 33829025 PMC8019728

[19] Michelozzi P de'Donato F Scortichini M De Sario M Noccioli F Rossi P. Mortality impacts of the coronavirus disease (COVID-19) outbreak by sex and age: rapid mortality surveillance system, Italy, 1 february to 18 april 2020. Eurosurveillance. (2020) 25:2000620. 10.2807/1560-7917.ES.2020.25.19.2000620 32431289 PMC7238743

[20] Dorrucci M Minelli G Boros S Manno V Prati S Battaglini M. Excess mortality in Italy during the COVID-19 pandemic: assessing the differences between the first and the second wave, year 2020. Front Public Health. (2021) 16:927. 10.3389/fpubh.2021.669209 34336767 PMC8322580

[21] Breslow NE Day NE. Statistical Methods in Cancer Research Volume II: The Design and Analysis of Cohort Studies, International Agency for Research on Cancer Scientific Publication No. 82. Lyon: *International Agency for Research on Cancer* (1986).3329634

[22] Ceccarelli E Minelli G Egidi V Lasinio GJ. Assessment of excess mortality in Italy in 2020-2021 as a function of selected macro-factors. Int J Public Health. (2023) 20:2812. 10.3390/ijerph20042812 36833508 PMC9956038

[23] Ciminelli G Garcia-Mandico S. COVID-19 in Italy: an analysis of death registry data. J Public Health. (2020) 42:723–30. 10.1093/pubmed/fdaa165 32935849 PMC7543414

[24] Riccardo F Ajelli M Andrianou XD Bella A Del Manso M Fabiani M. Epidemiological characteristics of COVID-19 cases and estimates of the reproductive numbers one month into the epidemic, Italy, 28 January to 31 March 2020. Euro Surveillance. (2020) 25:2000790. 10.2807/1560-7917.ES.2020.25.49.2000790 33303064 PMC7730489

[25] Rugge M Zorzi M Guzzinati S. SARS-CoV-2 infection in the Italian Veneto region: adverse outcomes in patients with cancer. Nat Cancer. (2020) 1:784–8. 10.1038/s43018-020-0104-9 35122051

[26] Serraino D Zucchetto A Dal Maso L Del Zotto S Taboga F Clagnan E. Prevalence, determinants, and outcomes of SARS-COV-2 infection among cancer patients. A population-based study in northern Italy. Cancer Med. (2021) 10:7781–92. 10.1002/cam4.4271 34551210 PMC8559499

[27] Mangone L Mancuso P Bisceglia I Rossi PG Chellini E Negro C. The impact of COVID-19 on new mesothelioma diagnoses in Italy. Thorac Cancer. (2022) 13:702–7. 10.1111/1759-7714.14296 35076994 PMC8888152

[28] Oddone E Bollon J Nava CR Consonni D Marinaccio A Magnani C. Effect of asbestos consumption on malignant pleural mesothelioma in Italy: forecasts of mortality up to 2040. Cancers. (2021) 13:3338. 10.3390/cancers13133338 34283067 PMC8267929

[29] Fazzo L Minelli G De Santis M Ceccarelli E Iavarone I Zona A. The epidemiological surveillance of mesothelioma mortality in Italy as tool for the prevention of asbestos exposure. Int J Public Health. (2023) 20:5957. 10.3390/ijerph20115957 37297561 PMC10252364

